# Notes of the Week

**Published:** 1921-04-23

**Authors:** 


### NOTES OF THE WEEK.

### HOSPITAL FOR SICK CHILDREN.

H.R.H. the Prince of Wales, President of the Hospital, will take the chair at the annual meeting of the Court of Governors of the Hospital for Sick Children, Great Ormond Street, W.C., to be held at the hospital on Tuesday, May 4, at 4 p.m.

### BRADFORD AGAIN.

While the controversy rages on the subject of the proposed new Municipal Hospital at Bradford, it is being said that the Health Service of this city is devoted to curing instead of preventing disease. During the meeting of the Boundary Extension Inquiry, Dr. T. E. Hill, County Medical Officer of Durham, stated that Bradford was badly scavenged, and has a persistently high infant mortality rate. Another Medical Officer said that the Health Service lacks imagination and development, the city has a great slum area in its very heart, and conditions exist there worse than in any mining village. The chances of prospective mothers dying in childbirth are twice as great in Bradford as anywhere else in the West Riding; this is largely owing to filthy homes. We do not think that the General Health Service can be so easily dissociated from an efficient hospital service, for the two things work side by side, and greatly help each other.

### LARGE GIFT TO THE WEST LONDON HOSPITAL.

One of the chief and most unassuming philanthropists of the Metropolis is Mr. Dan Mason, who at his own expense has provided and maintained the Chiswick Hospital, which is one of the most efficient cottage hospitals in the suburbs of London, but is seldom heard of because it never appeals for funds and relies entirely upon Mr. Mason's generosity. Mr. Mason is a director of the Chiswick Polish Co., which produces the well-known Cherry Blossom boot polish, and his latest benefaction is his gift of £22,000 towards the re-building of the out-patient department of the West London Hospital, where he is a member of the Board of Management, and in connection with which he defrayed a considerable part of the cost of building a home for the nurses.

### THE WELLHOUSE MUDDLE.

The position of Wellhouse Hospital has exercised for some time the Barnet Guardians and the local public. Dr. B. H. Stewart, the medical superintendent, has resigned, and Dr. Batty Shaw has withdrawn from the post of consulting physician because of disagreement among the local medical men. There are 204 beds in the hospital, a small number of paying patients, and the attempt to convert the institution into a public hospital has raised various vexed questions of which the paying patients and the desire to use the new buildings as a training-school for nurses are the chief. It is argued that the plan, which seeks to make the most of available hospital accommodation, will be expensive to the ratepayers and ineffective; but there are unfortunate factions at work, and the best of schemes can only be made successful by co-operation.

### A COMPETITION FOR ARCHITECTS.

It is announced that the Egyptian Government invite competitive designs for the new buildings of Qasr el 'Aini hospital and school at Cairo. The accommodation is for 1,225 beds. This competition will be conducted in two stages, the first open to all architects, the second restricted to twelve architects selected by the assessor from those submitting the best designs and nominated by H.H. Government. All applications for particulars should be addressed to H.E. the Minister of Public Works, Cairo, or to the Secretary, Royal Institute of British Architects, 9 Conduit Street, W. 1.

### THE NICHOLS PRIZE.

By the provisions of the will of the late Dr. R. T. Nichols the Royal Society will now offer triennially a prize of £250, open to British subjects, for the most valuable contribution on "The discovery of the causes and the prevention of death in childbirth from septicæmia." The Society will receive competing essays for the first award until, at latest, June 30, 1924. Work not published prior to June 30, 1921, will be eligible, and all information may be obtained from the Secretary of the Royal Society of Medicine, 1 Wimpole Street, W. 1. Although there is already considerable medical knowledge of this condition, and although mortality from it is less than it was, yet the establishment of the Nichols Prize should certainly help in stimulating research.

### WARD LET AS A FLAT.

Some time ago, when the house shortage was more acute, the Kensington Guardians made use of one of the empty wards in the workhouse by letting it to a man, his wife, and five children as a flat, at a rent of 11s. a week, including furniture. Apparently it was easier to let the ward than to become repossessed of it, and the Guardians have had to seek the assistance of the magistrate at the West London County Court to get rid of their tenant. He, however, is unable to find adequate accommodation elsewhere, and, as from the evidence given, it is apparent that the five children will soon be six children, the tenant is in some difficulty. He has, however, agreed to give up possession by May 21, or one month after the date of the augmentation of his family.

### A SUGGESTION TO MANCHESTER.

The interests of the Jews in Manchester are cared for by several bodies of their co-religionists, among which are the subscribers to the Board of Guardians for the relief of poor Jews in the city. Last December acute distress began to prevail, which the spread of unemployment will increase. The Board has issued a "crisis appeal" for £10,000. The consumptives present a difficult problem, for which a Jewish Health Committee under Dr. Haring has been formed. A health visitor has been appointed and a colony is being considered. The needs and charities of Manchester are so varied that the city might well take to heart the example of Liverpool and form a Council of Voluntary Aid to make the best of them. If something has been done in this direction it is not enough, and we should welcome further steps in this direction.

### CHARITIES COMPETING WITH INDUSTRY.

The question of the propriety of charities competing with industry in the fields of manufacture and public services is no new one. It has several times arisen in respect of charities for the blind, which through their workshops produce mats, baskets, and a number of other useful articles. Where it is plain that a number of afflicted people are enabled to help themselves, and that the work done is genuinely the result of their own endeavour, only finished or aided by others when the detail assistance is beyond their powers, and that they are not undercutting ordinary traders, there can be little objection to offer. But when, as recently announced at the annual meeting of a voluntary hospital, improvements and machinery have been provided for a laundry through a charge on charitable funds in order that "private laundry can be done at rates which compare favourably with those of outside laundries" (see p. 58) a question of fairness arises, because the phrase "compare favourably" can but have the meaning that competition as to price has been set up. It is true that the wording of the announcement implies that only subscribers to the charity are to be catered for; but even this restriction hardly meets the objections that some are almost certain to entertain against the principle involved.

### THE COST OF IN-PATIENTS.

Mr. C. F. Speyer, Secretary of St. Bartholomew's Hospital, Rochester, lately gave some interesting figures in answer to inquiries about patients' payments and the fees paid for disabled men. He said that the smallest patient's contribution was half-a-crown, and the largest £20. The Ministry of Pensions pays 2s. for each attendance by a disabled man at the out-patient department, and negotiations in regard to in-patients are taking place. The Ministry offered 9s. a day per patient, but the cost to the hospital is 11s. 6d.

### PARISH FUNDS FOR HOSPITALS.

The financial position of the Norfolk and Norwich Hospital has grown serious. With a total deficit of £28,000, patients' contributions have been started which average £60 a week. These are in charge of an almoner's committee, the duties of which absorb those of the previous admission and discharge committee. A monthly report of the work is presented by the almoner. Another successful development is that of parish funds, eighty of which have been started on the computation that a contribution of 2s. 3d. per head from each member of a parish served by the hospital would be a fair quota. These funds are fed from private subscriptions, workmen's contributions, and church collections, but each parish now knows what its total gift ought to be, and Mr. H. W. C. Drury, who is in charge of the scheme, has had a sympathetic hearing at each centre, so much so indeed that he thinks the scheme, with that started in the city, may yet save the situation. The hospital, in spite of a closed ward, is yet progressive, for its new Maids' Home should be fully in use by the middle of May, and we hope to publish a detailed description of it.

### A SOUTHAMPTON SUCCESS.

Colonel E. K. Perkins, C.B.E., chairman of the Royal South Hants and Southampton Hospital, announces the success of the recent effort to raise £10,000, with the gratifying result that the hospital is now free from debt. It is satisfactory to observe that this success is largely due to the hearty co-operation of the workers in such important firms as those of Messrs. Harland and Wolff, John I. Thornycroft, and Day and Summers. These men agreed to subscribe a penny in the pound, and the shipping companies also helped the movement. Southampton and Portsmouth have been centres where hospital finance has been depressing in the past; consequently this sign of a more vigorous spirit at Southampton is very encouraging, and may prove to be infectious.

### A MEDICAL OFFICER'S MOTOR-CAR.

Some discussion took place at the last meeting of the St. Pancras Board of Guardians regarding the decision of the Hospital Committee not to allow an assistant resident medical officer to keep a motor-car for private use on the premises of the institution, and eventually the matter was referred back for renewed consideration. In view of the nature of the duties of a medical officer at a large institution, coupled with the lack of social life, it would certainly appear desirable that arrangements should be made to allow of the medical staff securing adequate recreation. In the present case, we understand, it is not suggested there is lack of room to garage a motor-car, and unless some sound reason is advanced for refusal we hope that, on reconsideration, the Committee will grant the application.

### THE FIRST FRUITS OF A BOLD PLAN.

Many have watched with interest the result of the plan of Mr. Peter Wright, ex-Mayor of Newport, to raise £200,000 for the extension of the Royal Gwent Hospital. Last year the workmen's collections increased by £2,000, and we understand that the extension fund has now about £35,000 to its credit. The new mayor, Mr. W. A. Linton, J.P., and the committee first appointed are still at work on the scheme, which has justified the beginning of the new out-patient department and boiler-house. Richmond House, adjoining the hospital's pleasant grounds, has also been purchased, and is now occupied by seventeen members of the nursing and domestic staffs. The position, therefore, is improving, and if this can be maintained, not only will the income increase, but much will be done to realise Mr. Peter Wright's bold scheme for the extension fund.

